# Perfusion-ventilation CT via three-material differentiation in dual-layer CT: a feasibility study

**DOI:** 10.1038/s41598-019-42330-7

**Published:** 2019-04-09

**Authors:** Andreas P. Sauter, Johannes Hammel, Sebastian Ehn, Klaus Achterhold, Felix K. Kopp, Melanie A. Kimm, Kai Mei, Alexis Laugerette, Franz Pfeiffer, Ernst J. Rummeny, Daniela Pfeiffer, Peter B. Noël

**Affiliations:** 1 Department of Diagnostic and Interventional Radiology, Technical University of Munich, Munich, Germany; 2 Chair of Biomedical Physics, Department of Physics and Munich School of BioEngineering, Technical University of Munich, Garching, Germany; 3 Institute for Advanced Study, Technical University of Munich, Garching, Germany

## Abstract

Dual-Energy Computed Tomography is of significant clinical interest due to the possibility of material differentiation and quantification. In current clinical routine, primarily two materials are differentiated, e.g., iodine and soft-tissue. A ventilation-perfusion-examination acquired within a single CT scan requires two contrast agents, e.g., xenon and gadolinium, and a three-material differentiation. In the current study, we have developed a solution for three-material differentiation for a ventilation-perfusion-examination. A landrace pig was examined using a dual-layer CT, and three scans were performed: (1) native; (2) xenon ventilation only; (3) xenon ventilation and gadolinium perfusion. An in-house developed algorithm was used to obtain xenon- and gadolinium-density maps. Firstly, lung tissue was segmented from other tissue. Consequently, a two-material decomposition was performed for lung tissue (xenon/soft-tissue) and for remaining tissue (gadolinium/soft-tissue). Results reveal that it was possible to differentiate xenon and gadolinium in a ventilation/perfusion scan of a pig, resulting in xenon and gadolinium density maps. By summation of both density maps, a three-material differentiation (xenon/gadolinium/soft tissue) can be performed and thus, xenon ventilation and gadolinium perfusion can be visualized in a single CT scan. In an additionally performed phantom study, xenon and gadolinium quantification showed very accurate results (r > 0.999 between measured and known concentrations).

## Introduction

Computed tomography (CT) is an essential imaging modality for pulmonary diseases such as chronic obstructive pulmonary disease or pulmonary embolism^1,2^. During the last years, spectral CT has found widespread application in clinical routine and has been investigated intensively^3^. These systems acquire two x-ray spectra by applying different tube voltages. One solution - which requires the least hardware effort - is the sequential acquisition of two datasets at different tube voltages. Sequential acquisition can be performed either as two subsequent scans (rotate-rotate) or as subsequent rotations at alternating tube voltages (scan-scan). Due to the poor spatial-temporal registration between both acquisitions, contrast medium cannot be used and motion artefacts are of major concern^4^. In clinical practice today, two x-ray tubes with different peak kilovoltages (kVp) - so called dual-source CT (DS-CT) - or one tube with rapid kVp switching technology are used^5,6^. Here, spectral information is gained from two absorption measurements with normal CT detectors. To acquire spectral information with DS-CT systems, a scan must be designed accordingly before its start. With the described dual-energy CT (DE-CT) systems, material decomposition and material quantification became possible^7–9^. There are multiple applications for DE-CT^8–15^. Discrimination and quantification of materials such as iodine are of special interest as this is not possible with conventional CT systems^16^.

A different DE-CT concept was recently introduced into clinical routine^17,18^. This dual-layer CT (DL-CT) operates with one tube applying constant tube voltage. The detector consists of two layers: the upper layer absorbs low-energy photons and the lower layer absorbs high-energy photons^18^. Using this information, low- and high-energy images are obtained and by weighted summation, conventional images can be calculated. Spectral information can be extracted from perfectly aligned low- and high-energy detector data. In contrast to DS-CT systems, spectral information is acquired in every scan and thus a full retrospective spectral evaluation of CT data is possible. In addition, especially small iodine concentrations can be measured more accurately with DL-CT compared to DS-CT^19–21^.

In clinical routine, pulmonary function testing (PFT), e.g. spirometry, is used for indirect assessment of pulmonary ventilation. However, with PFT these parameters are evaluated globally for the whole lung. Regional changes are not considered, and pulmonary perfusion cannot be measured. With lung scintigraphy, regional changes in lung perfusion and ventilation can be imaged. However, diagnostic value is limited due to the lack of spatial resolution. For assessment of pulmonary ventilation patterns in patients with chronic obstructive pulmonary disease (COPD), pulmonary emphysema or bronchiectasis, xenon-ventilation can be analysed with DS-CT^22,23^. For this examination, patients inhale xenon (Xe) and then undergo DS-CT^24^. Resulting spectral images can be assessed regarding attenuation patterns, e.g. areas with outflow restriction can be identified and quantified^23^. Previous studies combined a xenon ventilation examination with a subsequent iodine-based perfusion examination on a DS-CT^25^. Here, two CT-scans are needed which are then combined using a dedicated software. One study showed that results of the ventilation/perfusion (V/Q) examination with DE-CT correlated significantly with PFT results in COPD patients^23^. To the best of our knowledge, there has been no study performing a ventilation and perfusion examination in only one DE-CT scan. Furthermore, xenon-ventilation imaging and xenon quantification has not yet been examined with DL-CT. Due to a k-edge close to iodine (xenon: 34.6 keV; iodine: 33.2 keV), an accurate quantification should be possible for xenon as well. However, this assumption was not yet evaluated in phantom studies.

With DE-CT, only one material (in clinical routine predominantly iodine) can be quantified as a decomposition with more than two materials is not possible. Special techniques such as the three-material decomposition using mass fraction are not - yet - used in clinical routine^26^. A quantification of more than two materials could become possible via a preselection regarding material distribution; e.g. via identification of lung tissue, subsequent material quantification of an inhaled contrast agent (xenon) and of an intravenously applied contrast agent (iodine or gadolinium) could be possible. Although this technique would not represent a “real” three-material decomposition, it could be one way to perform a V/Q-examination within a single CT-scan.

In the current study, accuracy of xenon and gadolinium quantification was examined in a thorax phantom model. The possibility of a simultaneous xenon-ventilation and gadolinium-perfusion CT examination using a tissue discriminating two-material decomposition was investigated in a pig-model.

## Results

### Quantification of Xenon

**Figure 1:**
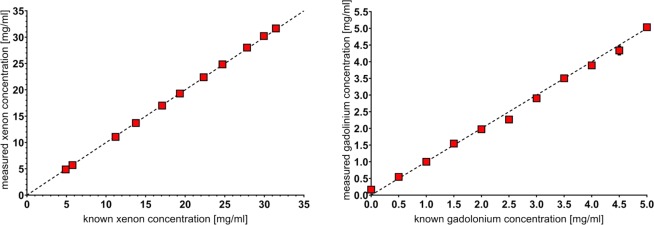
Measurement of xenon and gadolinium, compared to known concentrations. Xenon (**a**) and gadolinium (**b**) concentration measurements performed with a dual-layer spectral CT system. Red diamonds visualize the mean xenon density measured in three repetitive CT-scans (y-axis) vs. the known xenon concentrations (x-axis). Excellent correlations between scan results and measured concentrations were found (*r* > 0.999 for xenon and gadolinium). Note that some error bars cannot be visualized due to small values.

**Figure 2:**
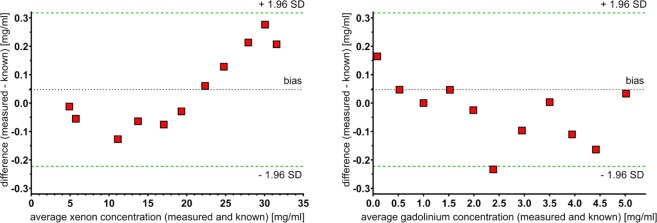
Bland-Altmann plots of the measurements. For xenon (**a**), a maximum error of 0.28 mg/ml (1.2%) was found for the 29.9 mg/ml sample. For all other concentrations, errors of less than 0.2 mg/ml are found. For gadolinium (**b**), errors of 0.23 mg/ml or less were found for all measurements. The highest percentage errors (9.4%/9.3%) were found for 0.5/2.5 mg/ml. For every other sample, the percentage error was 5% or lower.

Measured volume of the xenon bottle was 989 ml (±2 ml). Xenon quantification from material decomposition into xenon and non-xenon based on spectral data was compared to the known xenon concentrations (Fig. 1a). A high correlation coefficient (r > 0.999) was found between the known and every measured xenon concentration. No significant difference was found between repeated scans (p > 0.05). An error of 2‰ was appraised in the measurement of the bottle volume. Material decomposition errors are calculated by the SD of the means of repetitive measurement in one concentration; hereby errors are less than 0.3% for each measurement (Figs 1a and 2a). The mean difference between measured and known xenon concentrations was below 1.2% (0.28 mg/ml) for all measurements.

### Quantification of Gadolinium

A high correlation coefficient (r > 0.999) was found between the known and every measured gadolinium concentration (Fig. 1b). No significant difference was found between repeated scans (p > 0.05). The mean difference between measured and known gadolinium concentrations showed a maximum of below 0.23 mg/ml for all measurements. Differences of less than 0.1 mg/ml were found for all measurements but 0, 2.5, 4 and 4.5 mg/ml. All relative deviations stayed below 5% (apart from 2.5 mg/ml (9.3%) and 0.5 mg/ml (9.4%) - Figs 1b and 2b.

### Animal experiment

**Figure 3:**
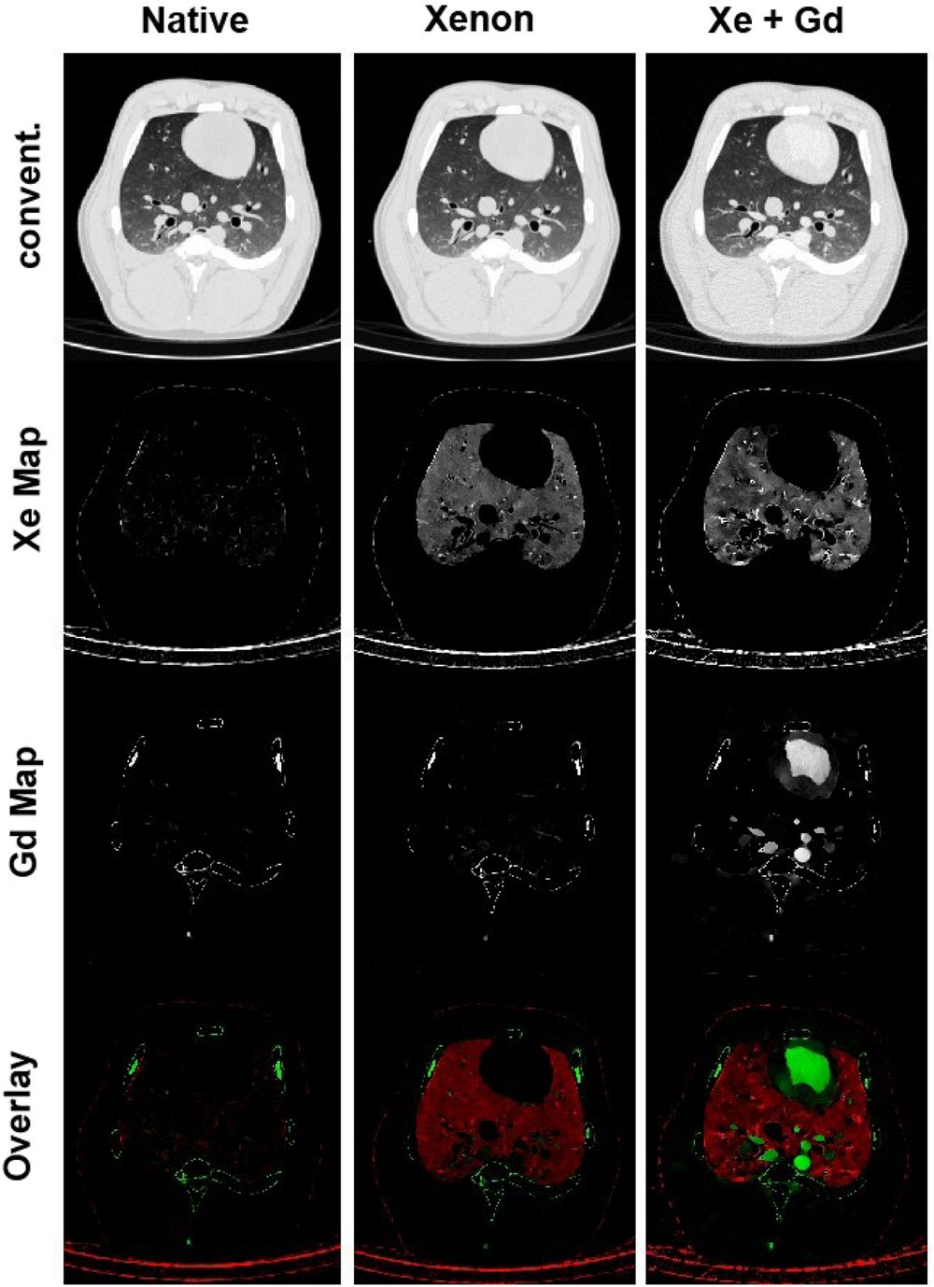
Axial plane visualizing the lung of the scanned pig. The columns show the native phase scan, the scan with xenon ventilation only and the scan with xenon ventilation and gadolinium perfusion combined. All images are visualized with the same window level and width. In conventional images (row one), no differentiation between xenon and gadolinium can be performed. With three-material differentiation, xenon and gadolinium alone (row two/three) can be visualized and quantified. By overlaying the xenon and the gadolinium density maps, a ventilation/perfusion examination can be visualized in one scan (row 4).

**Figure 4:**
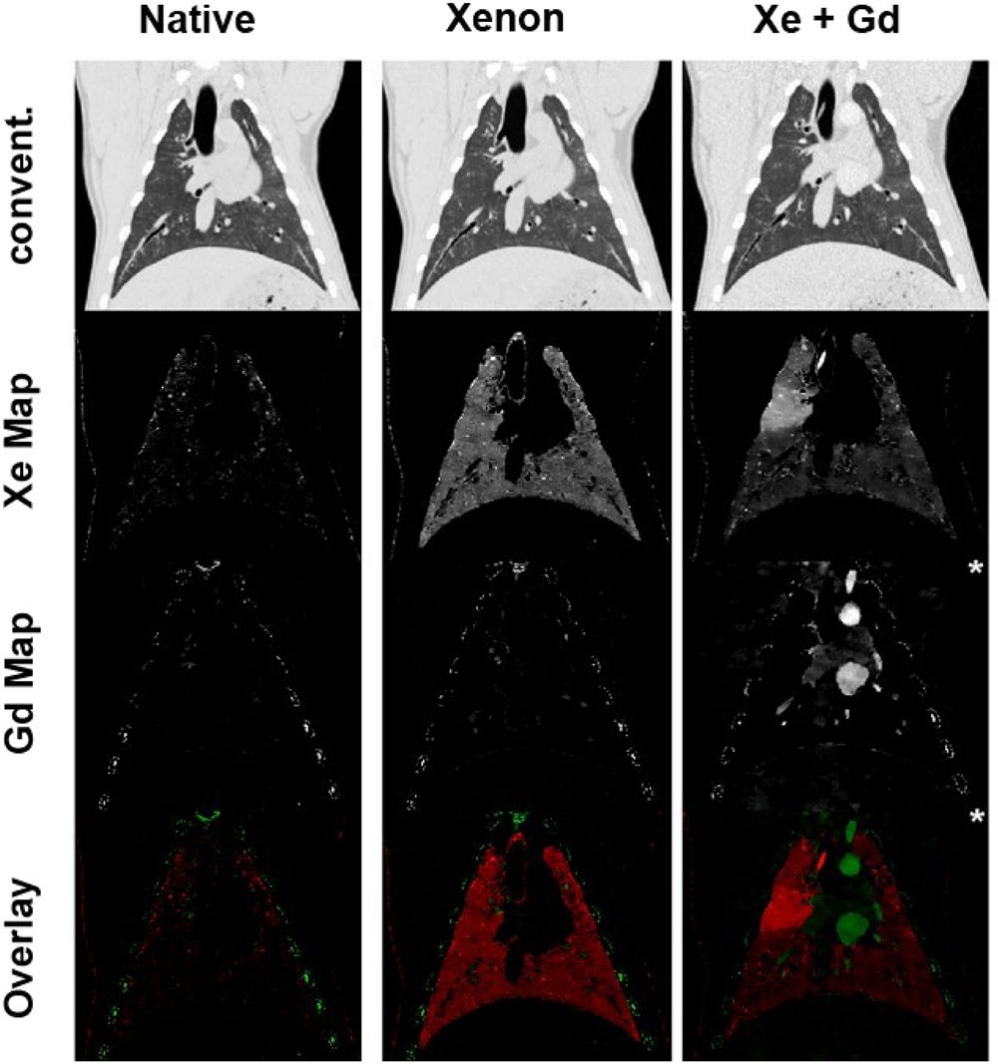
Coronal plane visualizing the lung of the scanned pig. The image order is the same as in Fig. 3. All maps are visualized with the same window level and width except star(*)-marked images, where the window is increased to adequately show the endotracheal tube in the tracheal bronchus. Note the increased xenon concentration in the right carinal lobe.

By solving the linear equation system on a pixel-by-pixel base for ${\rho }_{1}$ and ${\rho }_{2}$, 3D images for the material densities are generated and can be analysed in different anatomical planes. Conventional images and density maps are shown in Figs 3 and 4. Here, xenon and gadolinium can clearly be visualized and differentiated in the density maps. Gadolinium concentrations are relatively constant between central and peripheral pulmonary vessels. In the central vessels, concentrations of around 3 mg/ml can be found. In the peripheral vessels, slightly lower concentrations of 2.0–2.5 mg/ml can be found. In the aorta, a concentration of 3.3 mg/ml is observed. Within the lung parenchyma, gadolinium concentrations of up to 1.0 mg/ml are observed, representing gadolinium in small vessels. Hereby, the concentration of gadolinium showed an increase from the ventral to the dorsal parts of the lung, most likely due to atelectases.

Between scan 2 (xenon ventilation) and scan 3 (xenon ventilation and CTPA with gadolinium), a dislocation of the endotracheal tube into the tracheal bronchus is noted. The tracheal bronchus is the first bronchus coming of the trachea in pigs and ventilates the right cranial lobe. As for scan 3 (xenon ventilation and gadolinium CTPA) only the right cranial lobe is ventilated with xenon, an increased concentration of xenon in the right cranial lobe is expected compared to the remaining lung (here, only remaining xenon of the previous scan is present). This can clearly be visualized comparing xenon density maps of scans 2 and 3 (Figs 3 and 4). Distinct differences between the right cranial lobe and the remaining lung can also be noted when evaluating absolute xenon concentrations. In the right cranial lobe, concentrations of 2.2–2.8 mg/ml are observed. In the remaining lobes, concentrations of 0.3–1.2 mg/ml can be observed. Outside the lung, no xenon (0.0 mg/ml) is registered.

## Discussion

In this study, a three-material differentiation into xenon, gadolinium and soft tissue was performed with a dual-layer spectral CT. Additionally, accuracy of xenon and gadolinium quantification was evaluated.

High accuracy of material quantification could be observed with errors of measured material concentrations below 1.2% for all xenon test samples and below 5% for all gadolinium test samples (except the 0.5 and 2.5 mg/ml gadolinium sample). To the authors knowledge, there are no pre-existing studies determining the accuracy of xenon quantification in DL-CT systems. In theory, quantification should be possible accurately due to a k-edge close to iodine as iodine can be quantified very accurately^19^. For evaluation of ventilation patterns in CT scans, different regions of the lung are compared and thus an exact quantification of absolute xenon concentrations seems not essential. However, it must be guaranteed that differences in xenon concentrations (and thus different ventilation patterns) can be visualized reliably. Additionally, quantification could be of increasing interest if ventilation CT becomes a standard clinical procedure with standardized examination variables. Then, absolute values of xenon concentration could be used for the evaluation of pulmonary diseases such as emphysema. With the results of the present study, xenon quantification and the evaluation of pulmonary ventilation seem to be possible accurately and reliably. A previous study showed that gadolinium concentrations can be determined with an error of less than 10% in a clinically used DLCT spectral detector CT^27^. The results of the present study confirm these results, also showing high accuracies. In the present study, lower errors for very low gadolinium concentrations of 0.5 and 1.0 mg/ml could be shown, possibly due to improved firmware. Previous studies showed that DL-CT can accurately measure iodine, even for low iodine concentrations^19^. The current study confirms previous results and shows that also gadolinium can be measured accurately.

As described previously, between scan 2 (xenon ventilation only) and scan 3 (xenon ventilation and gadolinium CTPA), the endotracheal tube dislocated into the tracheal bronchus of the right carinal lobe. This was not planned during the study design; however, it increases the value of the present study. As between scans 2 and 3 xenon ventilation was performed, all applied xenon was applied to the right cranial lobe. This can clearly be visualized in scan 3 as xenon concentration in this lobe is significantly higher compared to the rest of the lung. Thus, it could be shown that a differentiation of ventilation patterns due to different xenon concentrations is possible *in vivo*. This affirms the results of the phantom model showing high accuracies of xenon quantification.

To the best of our knowledge, a three-material differentiation into xenon, gadolinium and soft tissue has not been performed previously with spectral CT. With DS-CT and DL-CT a direct three-material decomposition is not possible in clinical routine as only two energy levels can be detected and thus, the resulting information are only sufficient for solving the equitations for two materials. For a three-material decomposition, one additional information is needed. Previous studies have shown that a three-material decomposition is generally possible with these systems, however only for a mixture of solid materials or for solutions with a known material composition, as the composition is the needed third information^26^. One other way of performing a three-material decomposition is k-edge imaging with multi bin photon counting detectors^28^. To overcome this limitation in DE-CT, one workaround could be the approach presented in the present study. Here, two materials were quantified using a threshold for lung tissue (−200 HU) representing the third information, resulting in a three-material differentiation. For material-decomposition, voxels with lung tissue were differentiated into xenon and soft tissue whereas all other voxels were differentiated into gadolinium and soft tissue. Merging both two-material decompositions, one can differentiate between three materials. However, within one voxel, a three-material differentiation is not possible as each voxel is assigned to lung or soft tissue before material differentiation/quantification. As the ratio of air-filled alveoles and vessels is very high in the peripheral lung parenchyma, a differentiation in these segments seems not essential. In all areas with definable vessels, a three-material differentiation within one voxel is not needed as air and soft tissue cannot be present at the same time. With the described method, a ventilation/perfusion (V/Q) CT scan with three-material decomposition can be performed within one CT scan. A previous study described the possibility of performing a V/Q CT scan by performing a xenon-ventilation scan first and subsequently performing an iodine-based CTPA^25^. Another study also demonstrated the same principle in patients with suspected pulmonary embolism^24^. The latter study showed that V/Q imaging with DE-CT provides lung morphological and functional information and demonstrated a V/Q mismatch in patients with pulmonary embolism. However, the described method (xenon ventilation and subsequent CTPA) leads to a doubled radiation exposure compared to three-material differentiation. Additionally, longer examination durations are needed, i.e. five minutes between xenon-ventilation and CTPA in^24^. This is especially disadvantageous in patients with potentially life-threatening conditions like pulmonary embolism. Furthermore, artefacts or suboptimal overlays due to motion artefacts are possible as V/Q-images must be combined out of the ventilation and the perfusion scan. Compared to other procedures like V/Q-scintigraphy, a substantially increased resolution and a reduced examination time can be achieved with the method described in the current study.

Previous studies showed that CTPA could be improved using DS-CT^29^. However, the possibility of performing material decomposition into gadolinium and soft tissue for CTPA-scans was not evaluated. So far, only iodine-containing contrast agents are used for intravenously application in clinical CT scans. In the current study, gadolinium was used as intravenous contrast agent to evaluate the feasibility of a usage in the current setup, in V/Q examination and the capability of the used DL-CT system. As iodine is the established intravenous contrast agent for CT examinations, a further evaluation in the current study seemed obsolete. The current study confirms results of previous studies which showed that with DL-CT, accurate quantification of gadolinium (Gd) is possible^27^ and thus gadolinium could be used as an additional intravenous contrast agent^30^. As the present study shows, CTPA with gadolinium and generation of gadolinium density maps works satisfactorily with DL-CT, at least for visible vessels. A gadolinium concentration of 0.4 ml/kg body weight was used in the current study. This is below the maximum allowed concentration of 0.6 mg/ml body weight but is the maximum concentration used in our department in clinical routine. Thus, an increased contrast due to an increased concentration seems limited. With the described material decomposition into gadolinium and soft tissue, gadolinium CTPA could be further improved and could thus be used in clinical situations with contraindications (allergy or impaired renal function) for the use of iodinated contrast media. A diagnosis or rule-out of clinically relevant pulmonary embolisms in central and segmental pulmonary arteries should be well possible with the contrast achieved in the current study. However, CTPA with gadolinium must be evaluated further before it can be introduced into clinical routine.

The present study has some limitations. Firstly, only one pig-model was evaluated for this proof-of-concept study, so there are no additional measurements confirming the given results. However, the obtained measurements show that the described method for three-material differentiation works *in-vivo*. Secondly, a”real” three-material decomposition is not possible with dual-energy spectral CT. The described method is a way to overcome this limitation, however a presumption (lung tissue or other tissue) must be made before the both following two-material differentiations can be performed. For other regions like the abdomen, this three-material differentiation could not be used as no presumption regarding tissue affiliation is possible. Thirdly, xenon and gadolinium quantification cannot be tested *in vivo*. However, phantom evaluation shows that these quantifications are possible accurately and reliably.

In conclusion, the present study was the first to illustrate the possibility of performing a V/Q-examination during one CT scan in an animal model using a novel method of three-material differentiation in DL-CT. This could result in increased diagnostic value and decreased radiation exposure of V/Q-scans. Additionally, it could be shown that gadolinium-CTPA can be performed with DL-CT and that xenon and gadolinium quantification works accurately and reliably in DL-CT.

## Methods

### Quantification of xenon

To verify the accuracy of xenon density maps, a test series of varying xenon concentrations was scanned and investigated. Xenon 4.0 with 99.99 vol% xenon (Westfalen, Münster, Germany) was used for this purpose. The initial xenon concentration of 31.5 mg/ml was lowered stepwise down to 4.9 mg/ml by connecting the bottle to a precision control valve. The exact xenon concentration was calculated via measurement of the difference in weight (equalling the drained xenon mass) between scans with an analytical balance (precision up to 0.01 g) divided by the bottle volume. The exact volume of the bottle was measured using a volumetric measurement performed with micro CT system (Phoenix v|tome|x s 240, GE, Boston, MA, USA). A three-dimensional (3D) region growing algorithm was used to evaluate the exact bottle volume. Knowing the real xenon concentration (measured via weight and bottle volume), quantification of xenon in DL-CT was evaluated. For every xenon concentration, three independent scans of the bottle with a clinical standard dose (120 kVp, 100 mAs, CTDI_vol_ of 9 mGy, DLP of 70.9 mGy*cm) and one high dose scan (120 kVp, 1000 mAs, CTDI_vol_ of 90.3 mGy, DLP of 711 mGy*cm) were performed for every xenon concentration. Examined xenon concentrations were 4.90, 5.76, 11.19, 13.76, 17.08, 19.33, 22.33, 24.71, 27.81, 29.94 and 31.45 mg/ml.

### Quantification of gadolinium

To assess the accuracy of gadolinium quantification, conical centrifuge tubes (Greiner, Merck KGaA, Darmstadt, Germany) filled with a mixture of water and gadolinium contrast agent (Dotarem, Guerbet AG, Zürich, Switzerland) were investigated in the DL-CT scanner following the same acquisition parameters as for xenon quantification. Gadolinium concentrations of 20, 15, 10, 5.0, 4.5, 4.0, 3.5, 3.0, 2.5, 2.0, 1.5, 1.0. 0.5 and 0 mg/ml were pipetted by labour experienced staff (precision of 0.1 mg/ml). For evaluation of gadolinium quantification, the highest concentration (20 mg/ml) was used for the calibration of the mass attenuation coefficient.

### Animal experiment

#### CT image acquisition

A female landrace pig (50 kg) was examined using a DL-CT (IQon Spectral CT, Philips Healthcare, Best, the Netherlands). CT image acquisition was performed with the animal under deep general anaesthesia with endotracheal intubation and controlled ventilation. All animal procedures were approved by local authorities and were executed conforming to the ‘Guide for the Care and Use of Laboratory Animals’. Pre-anaesthesia sedation was performed with an intramuscular injection of Azaperon (2.0 mg/kg), Atropin (0.02 mg/kg) and Ketamin (15 mg/kg). General anaesthesia was initiated by the injection of Propofol (1%) by effect. After endotracheal intubation maintenance of anaesthesia was achieved by continuous injection of propofol 2% with bolus application of Fentanyl.

For all scans, the animal was positioned in supine position in the centre of the gantry. All CT scans were performed during endinspiratory breath-hold. Firstly, a native CT scan was performed. After inhalation of 100% Xenon (Westfalen AG, Münster, Germany) over 10 seconds, another CT scan was performed. Afterwards, a test bolus of gadolinium (Dotarem, Guerbet AG, Zürich, Switzerland) was injected intravenously to evaluate the circulation time. After ventilation of 100% xenon for another 10 seconds, a routine CT Pulmonary Angiography (CTPA) was performed with 20 ml of gadolinium (0.4 ml/kg) followed by a 50 ml saline chaser at a flow of 4 ml/s using a dual syringe injection system (Stellant, MEDRAD, Inc., Indianola, PA, USA).

For every scan, a tube voltage of 120 kVp with an exposure of 150 mAs was used. The CT Dose Index (CTDI_vol_) recorded in the dose protocol generated by the scanner was 13.5 mGy per scan, resulting in a DLP of 726 mGy*cm. The field of view was 350 mm with a pitch of 0.984, a rotation time of 0.33 seconds and a 64 × 0.625 mm detector configuration. Image matrix was 512 mm × 512 mm.

Spectral Base Image (SBI) data was reconstructed using a standard filter B, spectral level 2 and an axial slice thickness of 0.9 mm. The spectral level reconstruction parameter defines the smoothing strength of the reconstruction kernel. SBI datasets contain information on energy dependent absorption extracted with dual-layer detector technology. This information can be used to create virtual monochromatic (MonoE) images at energy levels from 40 to 200 keV using the vendor specific spectral software (IntelliSpace Portal v. 9.0.1, Philips Healthcare, the Netherlands); these images show Hounsfield Units (HU) information like images from a monoenergetic x-ray source^11^.

#### Image post-processing

An in-house developed algorithm was implemented to generate xenon and gadolinium density maps by applying a two-material decomposition on the spectral information from the MonoE images. In regions with lung tissue, a two-material decomposition into xenon and soft tissue was performed and in all other voxels, a two-material decomposition into gadolinium and soft tissue was achieved. This resulted in a three-material differentiation into gadolinium, xenon and soft tissue. The threshold defining the affiliation of a voxel to the lung tissue was set to −200 HU in the MonoE 100 keV image. Choosing this threshold seemed to be a good trade-off to separate between lung tissue with high xenon concentration and surrounding soft tissue. Due to similar material property, bone can hardly be differentiated from gadolinium when performing a material decomposition. Thus, the gadolinium density maps not only highlight gadolinium but also bone tissue. Therefore, bones (rips and vertebrae) were manually extracted from the gadolinium map.

The workflow of decomposing two linear independent MonoE images into xenon or gadolinium maps starts with the conversion from Hounsfield Units into attenuation coefficients using the formula:

1 $$\mu (E)=\frac{HU(E)\ast {\mu }_{water}(E)}{1000\,[HU]}+{\mu }_{water}(E)$$

Where ${\mu }_{water}(E)$ is the attenuation coefficient of water at energy $E$ (obtained from the United States National Institute of Technology and Standards (NIST) X-Ray Mass Attenuation Coefficients Database^31^). $HU(E)$ is the HU at energy $E$ given in the MonoE image. The linear equation system for the image-based material decomposition can then be written as follows:

2 $$\mu ({E}_{1})={\rho }_{1}{(\frac{\mu }{\rho })}_{1}({E}_{1})+{\rho }_{2}{(\frac{\mu }{\rho })}_{2}({E}_{1})$$

3 $$\mu ({E}_{2})={\rho }_{1}{(\frac{\mu }{\rho })}_{1}({E}_{2})+{\rho }_{2}{(\frac{\mu }{\rho })}_{2}({E}_{2})$$

$\mu (E)$ is the measured attenuation coefficient at energy ${E}_{1}$ (100 keV) or ${E}_{2}$ (200 keV), ${\rho }_{1}$ and ${\rho }_{2}$ are the densities of xenon and soft tissue or gadolinium and soft tissue and ${(\frac{\mu }{\rho })}_{1/2}(E)\,\,$are the known mass attenuation coefficients of the corresponding materials at energy ${E}_{1}$ or ${E}_{2}$.

Mass attenuation coefficients of xenon gadolinium and soft tissue are taken from the NIST X-Ray Mass Attenuation Coefficients Database^31^.

### Statistical analysis

Statistical analysis was performed by dedicated software packages (SPSS, IBM, USA; Excel 2016, Microsoft, USA). Continuous data are expressed as arithmetic mean ± SD. Spearman correlation *r* was calculated to assess correlation between measured and nominal xenon and gadolinium concentrations. Wilcoxon Signed-Rank Test was used to test for statistical difference between repetitive scans or between normal- and high dose scans. Measured and nominal xenon and gadolinium concentrations are shown in a cartesian coordinate system as well as in a Bland-Altman plot, respectively.

### Ethical approval

All animal procedures were approved by local authorities (55.2-1-54-2532.0-75-15, Regierung von Oberbayern, München, Germany) and were carried out in accordance with the ‘Guide for the Care and Use of Laboratory Animals’.

## Data Availability

The datasets generated during and/or analysed during the current study are available from the corresponding author on reasonable request.
